# Migration of Phthalates and Bisphenol A from Polyethylene Terephthalate Bottles into Beer During Storage at Controlled Temperatures

**DOI:** 10.3390/foods14152689

**Published:** 2025-07-30

**Authors:** Krešimir Mastanjević, Brankica Kartalović, Dragan Kovačević, Vinko Krstanović, Kristina Habschied

**Affiliations:** 1 Faculty of Food Technology, Josip Juraj Strossmayer University of Osijek, 31000 Osijek, Croatia; kmastanj@ptfos.hr (K.M.); dkovac@ptfos.hr (D.K.); vkrstano@ptfos.hr (V.K.); 2 Institute Biosens, Zorana Đinđića 1, 21000 Novi Sad, Serbia; brankica.kartalovic@biosense.rs

**Keywords:** migration, microplastic-related chemicals, GC-MS, PET

## Abstract

PET (polyethylene terephthalate) bottles contain different chemicals that can act as endocrine disruptors. Phthalates and bisphenol A can be found in various foods and beverages packaged in PET packaging or aluminum cans. For some phthalates, the European Union has established specified tolerable daily intakes for humans. This study aimed to establish the changes, types of phthalates (dimethyl phthalate, diethyl phthalate, diisobutyl phthalate, dibutyl phthalate, bis(2-ethylhexyl) phthalate, di-n-octyl phthalate), and bisphenol A concentrations in beer packaged in PET bottles and stored at two temperatures (4 °C and 20 °C) for four months. Beers were obtained from a local brewery after packaging into PET bottles and stored at the designated temperatures. GC-MS analysis was performed to determine phthalates and bisphenol A. Obtained data show that beers packaged in PET bottles can contain significant amounts of bisphenol A, and that their concentration increases with storage time. Phthalates were also identified in the samples, with the highest concentration of bis(2-ethylhexyl) phthalate found in the sample kept at 20 °C after 1 month of storage, sample P5; this concentration was 164.814 µg/L. BPA was recorded with the highest concentration in sample P11, which underwent 4 months of storage at a temperature of 20 °C.

## 1. Introduction

Chemicals such as phthalates (PAEs) and bisphenol A (BPA) have been applied in different industries, but they are especially utilized in plastic production. The polymer production industry uses them as plasticizers due to their characteristic properties to elasticize plastic mixtures [1,2]. Polyvinyl chloride (PVC), polyethylene terephthalate (PET), polyvinyl acetate (PVA), and polyethylene (PE) are commonly added to plastic mixtures to improve and modify the physical and chemical properties of plastic, providing properties such as extensibility, elasticity, and workability [1,3,4]. Plasticizers are common ingredients in general use products (toys, personal care and household products, car cosmetics, solvents, adhesives, glues, pesticides, food packaging, medical devices, electronics, tubing, and building materials) [5,6,7].

Plasticizers such as phthalates are known to be bio-, photo- and anaerobic-degradable, meaning that they cannot withstand environmental conditions [8,9]. The length of the alkyl groups dictates variability regarding water solubility. Longer chains have distinct lipophilicity and this poses a significant risk, especially in the packaging of fatty foods, fish, meat, dairy products, and vegetable oils that are rich in oils and fats and can act as solvents for plasticizers, enabling the migration of these polymers from food packaging to food [10]. Phthalates enter the gastrointestinal tract and then the rest of the body most commonly through food or beverages because they tend to dissociate from plasticized materials into food [11]. There are many sources of contamination from phthalates in the food production chain. This can occur via any of the unit operations and through transport, but also while using PVC gloves for food handling. They are added in printing inks or adhesives used on food wrappers, and thus, it is inevitable for them to migrate to food [11]. Some research reported phthalates in alcoholic beverages, but also strong alcoholic drinks [10,11,12,13,14]. Since phthalates are composed of phthalic acid esters with two ester groups in the ortho position, variations between phthalates become notable due to different alcohols that form the phthalic acid esters in the esterification reaction. Phthalates display high density, low solubility in water, but high solubility in organic solvents [15], which is why they are a potential threat in alcoholic beverages packages in PET bottles or aluminum cans whose inner surface is coated in plastic material. Alcohol content in beer can range from 3.0% to 8.0% (*v*/*v*), or even higher for certain types of beer. The majority of beer packaged in PET bottles contains about 5% alcohol (*v*/*v*) which is still a significant amount of alcohol to aid phthalate migration from packaging to beer. Besides the possibility of phthalates transfer to beer via direct contact (food packaging), there are many different pathways which can enable the chemicals to enter food, or in this case, beer [16]. For example, phthalate contamination may come from plastic gaskets, lids, and stoppers, as well as tetra packs, cans [17,18], and bottles, depending on how the product is processed, stored, and shipped. During the whole process of brewing and fermentation, maturation, and bottling, beer is in contact with plastic tubes and joints, stored in large containers for a certain period that may last up to several weeks. These stages can contaminate the product even before it reaches the packaging material.

Bisphenol A is a somewhat lipophilic molecule in its free form; however, through the process of conjugation (a biochemical process by which a substance becomes more soluble in water), bisphenol A becomes more hydrophilic [19]. It enters the human body mainly through migration from water bottles, food cans, the heating of plastic utensils, and from resin-based dental fillings. Dietary intake of bisphenol A is considered the most serious exposure to bisphenol A because it affects different age groups, including newborns, a particularly vulnerable age group. Dietary exposure to bisphenol A occurs over a long exposure period [20].

PET bottles are easier to transport, lighter than glass, possess good barrier properties regarding gas permeation, and are significantly lower in cost [21]. However, beer quality declines much quicker in PET bottles, reducing the storage time to 3-4 months. Recent studies have reported the appearance of different microplastic (MP) materials or chemicals related to MP. Since MP is thought to be a health-risk material, more and more research focuses on establishing its effect on human and animal health. The focal point is the reduction of MP particles and related chemicals in foodstuffs and the environment [22,23,24,25,26]. MP particles are known vectors for chemicals such as phthalates and bisphenol A. Namely, chemicals bond to MP particles and, as such, enter human or animal bodies [27].

Tolerable daily intakes for humans for bis(2-ethylhexyl) phthalate (DEHP), dibutyl phthalate (DBP), benzyl butyl phthalate (BBP), and bis(2-ethylhexyl) adipate (DEHA) have been set by the European Food Safety Authority (EFSA). The maximum levels of phthalates in food and beverages have not been prescribed by EU legislative bodies. There are only regulations on the migration of certain phthalates from the food contact material to the food. These amounts are small, with maximum residue limits being 1.5 mg/kg for DEHP, 0.3 mg/kg for DBP, 30 mg/kg for BBP, and 18 mg/kg for DEHA [27,28]. Since December 2024, BPA has been banned in food contact materials. Namely, the European Commission acknowledged EFSA’s (European Food Safety Agency) opinion on BPA. Setting the limit values on the amount of a chemical that may migrate from food packaging into food is the next step that the European Commission and Member State representatives will have to undertake [29,30,31]. Research on beer packaged in PET bottles is scarce, especially regarding chemicals and MP. MP-related chemicals are a topic that has recently gained attention and is important, especially since PET packaging occupies a significant share of the market.

Croatia has a significant amount of beer packaged in PET. Specifically, about 30% of beer is packaged in PET bottles [32]. Thus, this research intends to provide data about MP-related chemicals, such as phthalates and bisphenol A, in beer packaged in PET bottles and to evaluate the influence of temperature and storage time on the migration of these chemicals into beer. The novelty of this research is the screening the existing phthalates and BPA transferring to beer from PET packaging and the assessment of the optimal temperature and time for minimal transfer to occur.

## 2. Materials and Methods

### 2.1. Sample Preparation

Lager beer samples (22 bottles), freshly packaged in PET packaging (2 L), were obtained from the local brewery. GC-MS analysis was conducted to determine phthalates and bisphenol A content. All samples were produced in the same batch and stored in PET packaging from the same tank. Samples were stored in two environments; ten bottles were kept at 4 °C, with no exposure to daylight until analysis. The other ten bottles were stored in a chamber at a controlled temperature of 20 °C, again with no exposure to daylight. The first two samples were taken as the zero sample for both sampling groups. Other samples were analyzed in duplicates as well. Sampling was performed according to the plan described in Table 1.

Each sample was freshly opened, and the sample was excluded using a glass pipette (20 mL) and stored in a glass bottle (50 mL) with a glass cap. All glassware was rinsed with acetone and left to dry before use.

### 2.2. Determination of Phthalates

Phthalates were determined using GC-MS. Samples were prepared as described by Habschied et al. [33], and the analysis was performed in duplicates. Further analysis was performed according to the QuEChERS (Quick Easy Cheap Effective Rugged Safe) procedure [34]. Before analysis, calibration of the instrument was performed as described by Habschied et al. [33]. Table 2 presents limits of detection (LOD), quantification (LOQ), and R^2^ for the analyzed compounds.

### 2.3. Determination of Bisphenol A

In this study, a standard bisphenol A (BPA) solution (Sigma Aldrich, St. Louis, MI, USA) was used. The BPA solution was prepared at a concentration of 1 mg/mL. BPA solutions 0.005, 0.01, 0.1, 0.05, and 0.5 µg/mL were prepared by dilution in n-hexane (HPLC grade, Carlo Erba, Milan, Italy) and then stored in vials at −20 °C. To avoid cross-contamination with reagents, materials, and laboratory equipment, all glassware was subjected to a radical cleaning procedure. Soaking of glassware in acetone was conducted, and then it was dried at 140 °C for at least 4 h. All solvents used in the analysis were first tested for the presence of BPA by GC–MS analysis. The used ultrapure water was obtained by a Milli-Q system (Millipore, Bedford, MA, USA).

A total of 5 ml of the degassed sample was transferred to a glass tube with the addition of 5 mL ACN and 5 mL water as described by Habschied et al. [33].

BPA was identified via the comparison of the RT (retention time) of the peaks and target ions with those obtained from a standard of BPA.

The amount of BPA in the blanks was lower than the LOQ. Analysis was performed in splitless mode. The carrier gas was helium (*v* = 35.698 cm/s and *p* = 7.0 psi). The determination was performed at a constant flow rate.

### 2.4. Statistical Analysis

Statistical data were provided using analysis of variance (ANOVA) and Fisher’s least significant difference test (LSD) by Statistica 13.1. (TIBCO Software Inc., Palo Alto, CA, USA). The least statistical significance set to *p* < 0.05.

## 3. Results and Discussion

The results show that the values of the mentioned physicochemical parameters (Table 3) did not change much during the beer storage period.

**Table 3:** Results of physical–chemical analysis during storage.

Sample	Storage Time	Original Extract (°Plato)	Alcohol (mL/100 mL)	pH	Color (EBC)	Bitterness (BU)	Polyphenols (mg/L)
P1	0 days	10.97	4.4	4.3	6.4 ^i^	22 ^a^	84 ^d^
P2	14 days	10.97	4.4	4.3	7.2 ^h^	22 ^a^	89 ^c^
P3		10.97	4.4	4.3	7.3 ^h^	22 ^a^	90 ^bc^
P4	1 month	10.97	4.4	4.3	7.8 ^g^	21 ^b^	90 ^bc^
P5		10.97	4.4	4.3	8.0 ^f^	22 ^a^	90 ^bc^
P6	2 months	10.97	4.4	4.3	8.4 ^e^	20 ^c^	91 ^ab^
P7		10.97	4.4	4.3	8.9 ^d^	20 ^c^	91 ^ab^
P8	3 months	10.97	4.4	4.3	9.5 ^c^	20 ^c^	91 ^ab^
P9		10.97	4.4	4.3	11.0 ^b^	19 ^d^	92 ^a^
P10	4 months	10.97	4.4	4.3	10.0 ^c^	19 ^d^	91
P11		10.97	4.4	4.3	12.0 ^a^	19 ^d^	92 ^a^

Means within columns with different superscripts are significantly different (*p* < 0.05).

In this case, the basic wort extract, alcohol volume, and pH did not change at all, and the other three parameters had minor changes, with the largest differences in color, which is related to the aging of beer. Color changed significantly, and it seems that intense changes started in the first two weeks of storage, regardless of the temperature. An increase of 0.8 EBC was noted in the first two weeks of storage. The end values for color were much higher than the starting ones. Specifically, at the end of the experiment, after 4 months of storage at 4 °C, the color showed a 3.6 EBC unit increase concerning the starting 6.4 EBC. The last analyzed sample stored at 20 °C (P11) showed an almost twofold increase in color from the starting sample (6.4 EBC) to the ending 12.0 EBC. This, however, is an already recognized change and is in line with expectations of beer storage in PET packaging. Even though beer compounds oxidize during storage, and this is a completely natural process, beer color is often affected by enzymatic and nonenzymatic oxidation reactions, which result in the oxidation of polyphenols and the production of melanoidins as a result of the Maillard reaction [35,36]. Since the oxidation process is much more intense in PET packaging, storage time in such packaging is shorter than for other packaging materials (kegs, aluminum cans, or glass bottles) and is limited to 4 months.

Another physical–chemical parameter that changes during storage in PET is bitterness. Namely, changes in bitterness are also common during beer storage, and they usually imply a reduction, which is the case in this research as well [37,38]. According to the results, bitterness reduction was not influenced by storage temperature, but rather time of storage. Almost no difference was noted in beers stored at different temperatures.

Polyphenols show an increase during storage, also not related to the temperature, but with storage time. An increase in polyphenols is not usual during storage but can be explained by the presence of conjugates with the proteins in the fresh beer [39], since storage did not affect their concentration, or, as in this case, it aided their elevation.

Table 4 shows the results of the GC-MS analysis of phthalates in beer samples. Dimethyl phthalate and di-n-octyl phthalate were not detected in any of the samples and thus are left out of the table.

The highest recorded values were for DEHP, which can be explained by the wide use of this specific phthalate in the plastics industry. The highest concentration was found in sample P5, which was stored for 1 month at 20 °C, and was 164.814 µg/L, while for sample P4, which was stored at 4 °C for the same period, a value of 51.549 µg/L was determined. Sample P5 also had the highest values of diisobutyl phthalate (27.142 µg/L) and DBP (57.295 µg/L); on the other hand, it is the only sample in which no diethyl phthalate was found. The highest value for diethyl phthalate was in sample P1 and was 2.707 µg/L. The total value of all phthalates is the highest in sample P5, where it is 249.251 µg/L, and the lowest is 26.166 µg/L in sample P11. Samples stored for two weeks had a higher concentration of phthalates at a lower temperature. Diisobutyl and DBP after more than a month of storage, in all samples, had a higher concentration at a higher temperature. DBP had a higher value at a higher temperature after 2 and 4 months, and bis(2-ethylhexyl) phthalate after 1 and 3 months.

It is possible that the beer was contaminated with phthalates even before bottling because, according to research by Benc [40], it has been proven that phthalates can also potentially be transferred from malt to beer. There is also a high probability of cross-contamination during different stages of the process, and the reason for this is the ubiquity of phthalates in all types of plastic materials, such as pipette tips and the inside of bottle caps. Comparison with other alcoholic beverages has shown that beverages with a higher alcohol content have higher concentrations of phthalates. Alcohol content is a major factor in the migration of phthalates from packaging materials, pipes, and other equipment in the production process, probably due to their high solubility in ethanol [41].

The Republic of Croatia does not have a legally defined maximum permitted concentration of phthalates in packaging materials, soft drinks, and alcoholic beverages. In Croatia, legal acts currently regulate maximum permitted concentrations of phthalates only in products intended for infants. Therefore, additional research is needed that should focus on various potential factors and circumstances that may affect phthalate migration, including the following: pH value, type of preservative used in beverage production, level, type of alcohol, and the chemical composition of individual beverages.

Table 5 shows how much the concentration of bisphenol A changed during storage and at different temperatures in beers packaged in PET bottles.

It can be seen that in the initial sample that the concentration of bisphenol A was 0.020 µg/L, and during the first two weeks of storage at both temperatures, it increased to about 0.030 µg/L, i.e., in the sample stored at 4 °C after 14 days the concentration of BPA was 0.33 µg/L, and in beers stored at 20 °C the concentration was 0.029 µg/L. During the next two weeks, i.e., after a month of storage, the concentrations increased to 0.042 µg/L for beer stored at 4 °C and 0.060 µg/L. For sample P5, stored at 20 °C, this is a significant increase of 100%. The samples analyzed after two months of storage also show an increase in the concentration of bisphenol A in the samples, at both storage temperatures. The increase in BPA concentration continues during the next two months (total after 4 months) of storage, with the highest concentration recorded in sample P11, which was sampled after 4 months of storage at a temperature of 20 °C.

This research was motivated by the fact that beer packaging in PET bottles is common in the Republic of Croatia. In other countries, this is not as common, and there is a lack of literature data on the amounts of BPA in beers packaged in PET bottles. BPA is often detected in beers packaged in aluminum cans, with the main reason for its concentration in beers being the polymer coatings used to coat the inside of the can to prevent beer–aluminum contact. Research conducted by Habschied et al. [26] showed that phthalates are present in beers on the market. Some studies have confirmed the presence of BPA in beers packaged in aluminum cans in low concentrations (50–400 ng/L), which corresponds with the results in this study, although it is not the same packaging. According to some authors, although BPA was not added to the plastic from which PET bottles are produced [42,43], certain amounts of bisphenol A were found in PET bottles. Also, BPA migrates more easily in drinks packaged in bottles made of recycled plastic [43]. What is worrying, and confirmed in this research, is that BPA migrates into beer faster and in higher concentrations at higher temperatures. Namely, increasing the duration and temperature of storage, to above 40 °C, can increase the migration of BPA from PET bottles [44]. This information is important because beer is transported and, in many cases, stored under high temperature conditions, which obviously significantly affects the migration of BPA into beer.

The EU still does not have prescribed maximum values of phthalates in food and beverages. However, it does prescribe the amount of migration of certain phthalates from food contact material to the food. Maximum residue limits of 1.5 mg/kg are set for DEHP, 0.3 mg/kg for DBP, 30 mg/kg for BBP, and 18 mg/kg for DEHA [28,29]. The highest values obtained in this research for DEHP, 57.295 µg/L, and BBP, 27.142 µg/L, are still within the limits prescribed by the EU. However, the accumulated chemicals from different sources could pose a threat to human health.

Given that beer is a solution containing about 5% alcohol, it is likely that the migration of BPA into it will be more significant than in water; Dreolin et al. [43] have shown that the migration of BPA from bottles into an alcoholic solution (20% EtOH and 3% acetic acid) increases with increasing recycling rate.

## 4. Conclusions

According to the results of the research carried out in this paper, the presence of phthalates was detected in all beer samples. The highest concentration of phthalates was found in sample P5, which was stored for 1 month at 20 °C, and of the phthalates, DEHP had the highest value. It was found that after more than a month of storage, higher temperature leads to higher levels of diisobutyl and DBP.

With storage time and increased temperature during storage, there is an increase in the concentration of BPA in beer samples, and although the detected amounts in the samples seem minor, daily long-term exposure to this chemical can result in more serious consequences for human health. In any case, further research is needed to obtain a complete picture of how much BPA can be expected in various beer samples when exposed to variations in temperature and storage time.

There is a lack of studies with a sufficiently large number of samples that are focused on the determination of phthalates and BPA in beers on the market to draw reliable conclusions. This work has shown the presence of several different phthalates and BPA in beers, and although their health effects may not be serious due to the low levels found, the ubiquitous nature of these compounds in various sources can lead to cumulative exposure.

## Figures and Tables

**Table 1:** List of samples, temperatures, and exposure times.

Sample	Temperature (°C)	Time
P1	/	/
P2	4	14 days
P3	20	
P4	4	1 month
P5	20	
P6	4	2 months
P7	20	
P8	4	3 months
P9	20	
P10	4	4 months
P11	20	

**Table 2:** Limits of detection (LOD), quantification (LOQ), and R^2^.

	Dimethyl Phthalate	Diethyl Phthalate	Diisobutyl Phthalate	DBP	BBP	DEHP	Di-n-octyl Phthalate	Bisphenol A
LOQ (µg/mL)	0.0047	0.0010	0.0010	0.0010	0.0011	0.0011	0.0011	0.0011
LOD (µg/mL)	0.0014	0.0003	0.0003	0.0003	0.0003	0.0003	0.0003	0.0003
R^2^	0.997	0.999	0.997	0.998	0.993	0.997	0.996	0.996

Calibration ranges for all components were 0.005–0.5 µg/mL.

**Table 4:** Results of the GC-MS analysis of phthalates in beer samples expressed as (µg/L) and SD values.

Sample	Diisobutyl Phthalate	DBP	DEHP	Diethyl Phthalate
Unit	µg/L ± SD			
P1	2.707 ^a^ ± 0.022	10.079 ^c^ ± 0.109	6.425 ^e^ ± 0.389	44.761 ^d^ ± 0.128
P2	2.249 ^b^ ± 0.015	7.709 ^f^ ± 0.035	5.705 ^g^ ± 0.560	34.021 ^e^ ± 0.268
P3	0.355 ^g^ ± 0.011	4.907 ^j^ ± 0.030	5.132 ^i^ ± 0.017	31.895 ^f^ ± 0.634
P4	1.293 ^e^ ± 0.112	9.452 ^d^ ± 0.210	21.852 ^c^ ± 0.784	51.549 ^c^ ± 0.568
P5	0.000 ^i^ ± 0.002	27.142 ^a^ ± 0.120	57.295 ^a^ ± 0.634	164.814 ^a^ ± 0.078
P6	1.788 ^d^ ± 0.017	6.514 ^i^ ± 0.003	5.479 ^h^ ± 0.278	30.644 ^g^ ± 0.082
P7	2.218 ^b^ ± 0.019	6.767 ^g^ ± 0.378	8.317 ^d^ ± 0.178	18.492 ^h^ ± 0.186
P8	1.922 ^c^ ± 0.020	9.321 ^e^ ± 0.014	5.465 ^h^ ± 0.345	18.315 ^h^ ± 0.176
P9	0.406 ^f^ ± 0.004	10.835 ^b^ ± 0.078	28.181 ^b^ ± 0.632	126.669 ^b^ ± 0.584
P10	0.115 ^h^ ± 0.005	3.936 ^k^ ± 0.026	4.765 ^j^ ± 0.127	30.632 ^g^ ± 0.146
P11	1.737 ^d^ ± 0.018	6.647 ^h^ ± 0.038	5.961 ^f^ ± 0.548	11.821 ^i^ ± 0.008

Means within columns with different superscripts are significantly different (*p* < 0.05); SD—standard deviation.

**Table 5:** The amount that the concentration of bisphenol A changed during storage and at different temperatures in beers packaged in PET bottles, expressed as (µg/L) and SD values.

Sample	BPA µg/L ± SD
P1	0.020 ^i^ ± 0.003
P2	0.033 ^h^ ± 0.005
P3	0.029 ^g^ ± 0.012
P4	0.042 ^f^ ± 0.011
P5	0.060 ^d^ ± 0.019
P6	0.052 ^e^ ± 0.006
P7	0.061 ^d^ ± 0.003
P8	0.065 ^c^ ± 0.006
P9	0.066 ^bc^ ± 0.04
P10	0.068 ^b ±^ 0.017
P11	0.087 ^a^ ± 0.015

Means within columns with different superscripts are significantly different (*p* < 0.05); SD—standard deviation.

## Data Availability

The original contributions presented in the study are included in the article. Further inquiries can be directed to the corresponding author.
